# Who is a global health expert?

**DOI:** 10.1371/journal.pgph.0002269

**Published:** 2023-08-17

**Authors:** Chiamaka P. Ojiako, Lazenya Weekes-Richemond, Vuyiseka Dubula-Majola, Marie-Claire Wangari

**Affiliations:** 1 Women in Global Health-UK Chapter, London, United Kingdom; 2 Centre for Civil Society, University of KwaZulu-Natal, Durban, South Africa; 3 Women in Global Health-Kenya Chapter, Nairobi, Kenya; PLOS: Public Library of Science, UNITED STATES

## Introduction

In a previous blog post [1] we wrote that there is neither a universally accepted definition of global health expertise nor credentials to decide who a global health expert is. This is both a blessing and a curse. On the one hand, global health is an inherently interdisciplinary field and allows diverse roles and actors. On the other hand, the lack of consensus on what makes a global health expert has created global health experts by default, often based on nationality or ethnicity (i.e. elevation of whiteness as a marker of expertise) or country of residence, resulting in an uneven skew of global health “experts” from HIC, and often resulting in major decisions being made far away from where the real problems are and where the real expertise exists. Data clearly show that global health organizations are typically led by people with power and privilege in the Global North (with older, white men as the most dominant group), while only a tiny fraction of leadership roles or board seats are held by people from LMICs [2].

This skewed distribution perpetuates a history of colonialism and exploitation that elevates access to resources, whiteness, and branding as key factors for developing expertise rather than lived experience and competence. Furthermore, it has excluded health professionals in the Global South [3] and caused them to be hesitant about declaring their expertise even when they are experts, in the proper sense.

## Expertise is not limited to medical or health professionals

Global health evolved from colonial medicine, tropical medicine, and international health with a primary focus on medical and health problems [4]. Despite the crucial role of medical and health professionals, global health is more than medical care and extends to addressing other underlying drivers of health, social and political determinants, and non-health sectoral issues.

The COVID-19 pandemic showed us that merely brandishing evidence, facts, and science is not enough to ensure equity or improve outcomes. Culture, human behaviour, governance, law, politics, regulations, and institutional frameworks are equally key aspects of global health and require non-health professionals versed in health and their area of discipline.

And yet, we continue to put too much weight on only some of these experiences (i.e., doctors, scientists, etc). We must recognize that global health expertise is not limited to the medical, public health and other science-related professions. We must start valuing experts from other professions and experts who truly bring multi-disciplinarity to their practice.

## Expertise is not limited to academia or high-income nations

Despite the significant work and contributions of people from LMICs to global health research and program implementation, often they do not see themselves as global health experts given that global health is a field that is driven by HICs [5].

In the health research space, health professionals with academic accolades and a wide network of similarly credentialed professionals are more likely to be sought out as global health experts. HIC researchers have substantially greater access to research funding, end up publishing much more, and often get much of the academic credit, even when research is primarily conducted in LMICs. This power dynamic undervalues LMIC researchers with expertise and lived experiences, excludes them from authorship and relegates their knowledge and efforts to mere contributions or acknowledgements [6].

When the knowledge emanates from LMICs, it is often deemed unsophisticated or not rigorous enough to be recognized as evidence. Meanwhile, HIC researchers work with the knowledge acquired by LMIC experts, reap the benefits of the knowledge or discovery and gain recognition as global health experts [7]. Research funding organisations must address this via equitable funding models, including a more transparent and direct funding approach of LMIC researchers.

The current understanding of global health expertise is reflective of embedded elements of residual colonialism that considered white individuals as the only experts. This narrow construct of global health expertise excludes the diverse and lived experiences of health professionals working on the front lines and at the community level in LMICs [1, 8]. Limiting global health expertise to only academic qualifications and a few years of ‘field’ experience in the LMICs rubber stamps the self-proclaimed expert status [9]. It allows the absurdity of labelling individuals with no lived experience as experts while under-valuing front line health workers as mere health providers.

Uneven representation of global health experts in the LMICs is further enabled by the conferment of expertise based on appointments to serve on boards, expert committees, or technical groups. Invitations to serve on these committees are usually made to professionals serving on the management boards of global health organisations. With the poor representation of LMICs representation in global health organisations’ leadership positions, it reduces the chances of being conferred expertise through this pathway.

Undoubtedly, there are global health professionals from the HICs that are subject experts and genuinely seek to understand the environments they work in. However, the implicit benefit of the doubt that equates expertise to merely being from the HICs allows the less qualified to thrive [10].

## Advice for aspiring global health experts

While global health is a popular career path among young people today, there is also a growing acknowledgement of the inequities inherent in global health. We can take advantage of the discourse around decolonization of global health and a window to expand the makeup and vision of experts invited to the policymaking table. We should encourage early career researchers into the field, and here are some suggestions to consider for those contemplating entering global health.

First, understand what drives you. Deeply reflect on your motivation for aspiring to become a global health expert. Is it personal recognition? To ‘save people’? Higher salary? Influencing power over policy and/or decision-makers, gaining more funding from donors/grant awards, fostering public trust, power over people/teams/decision-makers, or a combination of these things? Understanding your motivation may help you in identifying the routes you wish to take to gain expert status. We advise against seeking to become a global health expert to satiate your desire for saviourism or travelling or experiencing new cultures. Instead, the key question should be, "Why does it matter to you that people in such-and-such communities are affected by such-and-such disease”?

Second, be humble and respectful of people’s lived experiences, as this can be worth more than qualifications or publications. Be intentional about including and recognising those from local communities, especially from underrepresented communities and LMICs, as global health experts.

Third, be comfortable being lifelong learners and open to new ideas and different viewpoints and be passionate about your chosen topic in global health. Be comfortable with the idea that you will be wrong and be humble to changing your point of view.

Fourth, balance your academic training with hands-on work experience in culturally diverse settings as a deep understanding and appreciation of cultural contexts is fundamentally important. Be a good listener, collaborator, and ally.

Fifth, identify role models to emulate, actively seek several mentors and note they don’t have to be older, or be based in the Global North. Conversely, you can learn valuable lessons from finding and learning from people who have the qualities you aspire to and do the work in a manner that resonates with you rather than focusing on formal mentorship.

Lastly, do the work–ensure you are contributing to improving lives–not in theory, but in reality.
